# Poly[bis­(acetonitrile-κ*N*)bis­[μ_3_-bis(tri­fluoro­methane­sulfonyl)­imido-κ^4^ *O*,*O*′:*O*′′:*O*′′′]dilithium]

**DOI:** 10.1107/S1600536811011561

**Published:** 2011-04-07

**Authors:** Daniel M. Seo, Paul D. Boyle, Wesley A. Henderson

**Affiliations:** a Department of Chemical and Biomolecular Engineering, North Carolina State Univerisity, Raleigh, NC 27695, USA; b Department of Chemistry, North Carolina State Univerisity, Raleigh, NC 27695, USA

## Abstract

In the title compound, [Li_2_(CF_3_SO_2_NSO_2_CF_3_)_2_(CH_3_CN)_2_]_*n*_, two Li^+^ cations reside on crystallographic inversion centers, each coordinated by six O atoms from bis(trifluoromethanesulfonyl)imide (TFSI^−^) anions. The third Li^+^ cation on a general position is four-coordinated by two anion O atoms and two N atoms from acetonitrile mol­ecules in a tetra­hedral geometry.

## Related literature

For the structure of LiN(SO_2_CF_3_)_2_, see: Nowinski *et al.* (1994) ▶. For a related structure of LiN(SO_2_CF_3_)_2_, see: Henderson *et al.* (2005 ▶); Davidson *et al.* (2003 ▶); Brouillette *et al.* (2002 ▶); Dillon *et al.* (2001) ▶. For the structure of CH_3_CN with lithium salts, see: Klapötke *et al.* (2006 ▶); Brooks *et al.* (2002 ▶); Yokota *et al.* (1999) ▶; Raston *et al.* (1989 ▶). 
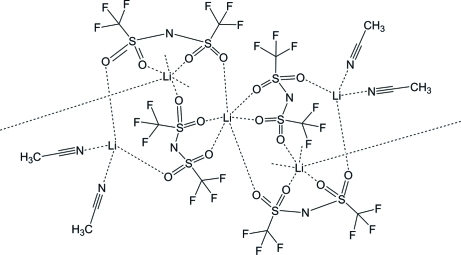


## Experimental

### 

#### Crystal data

[Li_2_(C_2_F_6_NO_4_S_2_)_2_(C_2_H_3_N)_2_]
*M* *_r_* = 656.29
Monoclinic, 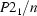

*a* = 10.8654 (2) Å
*b* = 11.0610 (2) Å
*c* = 19.1778 (3) Å
β = 90.8483 (10)°
*V* = 2304.58 (7) Å^3^
*Z* = 4
Cu *K*α radiation
μ = 5.16 mm^−1^
*T* = 110 K
0.40 × 0.20 × 0.15 mm

#### Data collection

Bruker–Nonius X8 APEXII diffractometer
Absorption correction: multi-scan (*SADABS*; Bruker, 2009 ▶) *T*_min_ = 0.232, *T*_max_ = 0.512
9937 measured reflections
3950 independent reflections
3482 reflections with *I* > 2σ(*I*)
*R*_int_ = 0.041

#### Refinement

*R*[*F* ^2^ > 2σ(*F* ^2^)] = 0.041
*wR*(*F* ^2^) = 0.108
*S* = 1.07
3950 reflections
348 parameters
H-atom parameters constrained
Δρ_max_ = 0.66 e Å^−3^
Δρ_min_ = −0.49 e Å^−3^

### 

Data collection: *APEX2* (Bruker, 2009 ▶); cell refinement: *SAINT* (Bruker, 2009 ▶); data reduction: *SAINT*; program(s) used to solve structure: *SIR92* (Altomare *et al.*, 1994 ▶); program(s) used to refine structure: *SHELXTL* (Sheldrick, 2008 ▶); molecular graphics: *ORTEP-3* (Farrugia, 1997 ▶); software used to prepare material for publication: *cif2tables.py* (Boyle, 2008 ▶).

## Supplementary Material

Crystal structure: contains datablocks I, global. DOI: 10.1107/S1600536811011561/si2342sup1.cif

Structure factors: contains datablocks I. DOI: 10.1107/S1600536811011561/si2342Isup2.hkl

Additional supplementary materials:  crystallographic information; 3D view; checkCIF report

## Figures and Tables

**Fig. 1.**
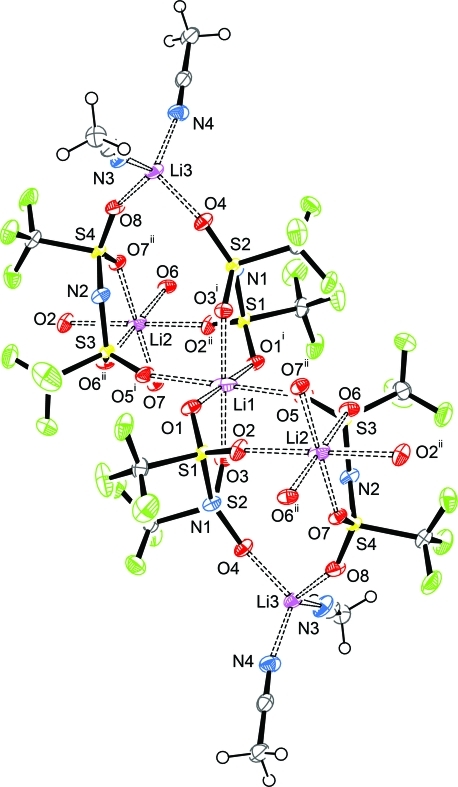
Molecular structure of the title compound. The thermal ellipsoids are shown at a 50% probability level. (Symmetric codes: (i)-x + 2, -y - 1, -z + 1; (ii) -x + 2, -y, -z + 1)

**Fig. 2.**
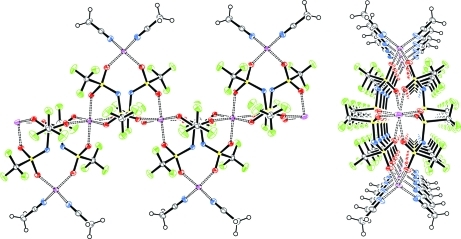
Schematic illustration of ion and solvent coordination for the title compound.

## supplementary crystallographic information

### Comment

The structure contains three symmetry independent Li^+^ cations. Two of these, Li1 and Li2, reside on crystallographic inversion centers and are each are coordinated by six O atoms from TFSI^-^ anions in a pseudo-octahedral coordination geometry. The third Li^+^ cation, Li3, sits at a general position and is four coordinate: two O atoms and two N atoms from acetonitrile molecules form a pseudo-tetragonal coordination geometry. There are two different TFSI^-^ anions which ligate the Li^+^ cations Li1 and Li2 by chelating a single lithium as well as bridging the Li1···Li2 sites. These two lithium sites are joined by two TFSI^-^ anions to form eight membered rings. The rings are formed using atoms {O1, O2} and {O5, O6}, while the axial coordination sites for Li1 and Li2 are occupied by O3 and O7, respectively. These rings form a polymeric chain which propagates along the [0 1 0] direction. Two of the coordination sites for the four coordinate Li3 atom are occupied by O4 and O8, thus providing a link between two TFSI^-^ ligands. The other two coordination sites are occupied by the N atoms from two different acetonitrile molecules. The methyl tails as well as the CF_3_ groups from the TFSI^-^ anions form the exterior of the polymeric chains.

### Experimental

LiTFSI was purchased from 3*M* and dried under high-vacuum at 393 K. Anhydrous acetonitrile (Sigma Aldrich, 99.8%) was used as-received. In a vacuum atmospheres (N_2_) glove box (< 5 p.p.m. H_2_O), LiTFSI (5 mmol) and acetonitrile (6 mmol) were sealed in a vial and the mixture heated on a hot plate to form a homogeneous solution. 2 ml of toluene was then added to the vial to dilute the mixture. Upon standing at 278 K in a refrigerator, colorless plate single crystals formed suitable for analysis.

### Refinement

The structure was solved by direct methods using the *SIR92* program. All non-hydrogen atoms were obtained from the initial solution. The structural model was fit to the data using full matrix least-squares based on *F^2^*. The calculated structure factors included corrections for anomalous dispersion from the usual tabulation. The structure was refined using the XL program from *SHELXTL*, and graphic plots were produced using the *ORTEP-3* program. Methyl hydrogens were introduced at idealized positions and were allowed to ride on the parent carbon atom with C—H = 0.98 Å and *U*_iso_(H) = 1.5 times *U*_eq_(C).

### Crystal data

**Table:**

[Li_2_(C_2_F_6_NO_4_S_2_)_2_(C_2_H_3_N)_2_]	*F*(000) = 1296
*M**_r_* = 656.29	*D*_x_ = 1.892 Mg m^−^^3^
Monoclinic, *P*2_1_/*n*	Melting point: 315.68 K
Hall symbol: -P 2yn	Cu *K*α radiation, λ = 1.54178 Å
*a* = 10.8654 (2) Å	Cell parameters from 4162 reflections
*b* = 11.0610 (2) Å	θ = 4.6–65.8°
*c* = 19.1778 (3) Å	µ = 5.16 mm^−^^1^
β = 90.8483 (10)°	*T* = 110 K
*V* = 2304.58 (7) Å^3^	Plate, colourless
*Z* = 4	0.40 × 0.20 × 0.15 mm

### Data collection

**Table:**

Bruker–Nonius X8 APEXII diffractometer	3950 independent reflections
Radiation source: fine-focus sealed tube	3482 reflections with *I* > 2σ(*I*)
graphite	*R*_int_ = 0.041
ω and φ scans	θ_max_ = 66.2°, θ_min_ = 4.6°
Absorption correction: multi-scan (*SADABS*; Bruker, 2009)	*h* = −12→12
*T*_min_ = 0.232, *T*_max_ = 0.512	*k* = −13→9
9937 measured reflections	*l* = −22→22

### Refinement

**Table:**

Refinement on *F*^2^	Primary atom site location: structure-invariant direct methods
Least-squares matrix: full	Secondary atom site location: difference Fourier map
*R*[*F*^2^ > 2σ(*F*^2^)] = 0.041	Hydrogen site location: inferred from neighbouring sites
*wR*(*F*^2^) = 0.108	H-atom parameters constrained
*S* = 1.07	*w* = 1/[σ^2^(*F*_o_^2^) + (0.0689*P*)^2^ + 0.1796*P*] where *P* = (*F*_o_^2^ + 2*F*_c_^2^)/3
3950 reflections	(Δ/σ)_max_ = 0.001
348 parameters	Δρ_max_ = 0.66 e Å^−^^3^
0 restraints	Δρ_min_ = −0.49 e Å^−^^3^

### Special details

**Table:**

Geometry. All e.s.d.'s (except the e.s.d. in the dihedral angle between two l.s. planes) are estimated using the full covariance matrix. The cell e.s.d.'s are taken into account individually in the estimation of e.s.d.'s in distances, angles and torsion angles; correlations between e.s.d.'s in cell parameters are only used when they are defined by crystal symmetry. An approximate (isotropic) treatment of cell e.s.d.'s is used for estimating e.s.d.'s involving l.s. planes.
Refinement. Refinement of *F*^2^ against ALL reflections. The weighted *R*-factor *wR* and goodness of fit *S* are based on *F*^2^, conventional *R*-factors *R* are based on *F*, with *F* set to zero for negative *F*^2^. The threshold expression of *F*^2^ > σ(*F*^2^) is used only for calculating *R*-factors(gt) *etc*. and is not relevant to the choice of reflections for refinement. *R*-factors based on *F*^2^ are statistically about twice as large as those based on *F*, and *R*- factors based on ALL data will be even larger.

### Fractional atomic coordinates and isotropic or equivalent isotropic displacement parameters (Å^2^)

**Table:**

	*x*	*y*	*z*	*U*_iso_*/*U*_eq_	
Li1	1.0000	−0.5000	0.5000	0.0241 (13)	
Li2	1.0000	0.0000	0.5000	0.0319 (15)	
Li3	1.2401 (4)	−0.2523 (4)	0.7428 (2)	0.0212 (8)	
S1	0.86669 (5)	−0.27813 (5)	0.57295 (3)	0.01533 (16)	
S2	1.02904 (5)	−0.40779 (5)	0.65910 (3)	0.01465 (16)	
O1	0.86025 (16)	−0.38148 (16)	0.52784 (9)	0.0198 (4)	
O2	0.88818 (17)	−0.16218 (16)	0.54296 (10)	0.0242 (4)	
O3	1.05935 (15)	−0.48372 (15)	0.60138 (9)	0.0189 (4)	
O4	1.12685 (17)	−0.37177 (16)	0.70505 (10)	0.0235 (4)	
N1	0.9472 (2)	−0.29426 (18)	0.64117 (11)	0.0189 (4)	
C1	0.7103 (2)	−0.2650 (3)	0.60816 (15)	0.0259 (6)	
F1	0.63021 (15)	−0.26086 (15)	0.55589 (10)	0.0322 (4)	
F2	0.70074 (18)	−0.1660 (2)	0.64600 (12)	0.0514 (6)	
F3	0.68438 (17)	−0.3600 (2)	0.64704 (11)	0.0514 (6)	
C2	0.9349 (3)	−0.5030 (2)	0.71598 (14)	0.0223 (5)	
F4	0.83885 (15)	−0.54657 (15)	0.68038 (9)	0.0322 (4)	
F5	0.89314 (17)	−0.43864 (16)	0.76878 (8)	0.0338 (4)	
F6	1.00206 (18)	−0.59308 (15)	0.74022 (10)	0.0387 (4)	
S3	1.17709 (5)	−0.22534 (5)	0.47597 (3)	0.01494 (16)	
S4	1.22060 (5)	−0.09504 (5)	0.59873 (3)	0.01427 (16)	
O5	1.11998 (18)	−0.34116 (16)	0.46985 (9)	0.0227 (4)	
O6	1.11351 (17)	−0.12146 (16)	0.44846 (9)	0.0211 (4)	
O7	1.11864 (15)	−0.01780 (16)	0.58177 (9)	0.0194 (4)	
O8	1.23617 (17)	−0.13043 (16)	0.66994 (9)	0.0223 (4)	
N2	1.23502 (19)	−0.21021 (18)	0.55127 (11)	0.0182 (4)	
C3	1.3146 (3)	−0.2392 (2)	0.42186 (15)	0.0237 (5)	
F7	1.28089 (18)	−0.24474 (16)	0.35552 (9)	0.0344 (4)	
F8	1.38759 (17)	−0.1450 (2)	0.43007 (10)	0.0463 (5)	
F9	1.3759 (2)	−0.3386 (2)	0.43789 (11)	0.0532 (6)	
C4	1.3576 (2)	−0.0027 (2)	0.58387 (14)	0.0202 (5)	
F10	1.36085 (16)	0.08703 (15)	0.62945 (10)	0.0336 (4)	
F11	1.35530 (15)	0.04254 (15)	0.52012 (9)	0.0305 (4)	
F12	1.45820 (14)	−0.06888 (15)	0.59205 (10)	0.0310 (4)	
N3	1.3951 (2)	−0.3487 (2)	0.75516 (13)	0.0278 (5)	
C5	1.4711 (2)	−0.4159 (2)	0.76885 (14)	0.0234 (6)	
C6	1.5678 (3)	−0.5024 (3)	0.78594 (15)	0.0285 (6)	
H61	1.6388	−0.4879	0.7562	0.043*	
H62	1.5371	−0.5847	0.7782	0.043*	
H63	1.5927	−0.4929	0.8350	0.043*	
N4	1.1814 (2)	−0.1550 (2)	0.82413 (12)	0.0289 (5)	
C7	1.1632 (3)	−0.0855 (2)	0.86648 (14)	0.0240 (6)	
C8	1.1392 (3)	0.0036 (3)	0.92008 (15)	0.0274 (6)	
H81	1.1588	0.0844	0.9025	0.041*	
H82	1.1904	−0.0138	0.9614	0.041*	
H83	1.0521	0.0004	0.9326	0.041*	

### Atomic displacement parameters (Å^2^)

**Table:**

	*U*^11^	*U*^22^	*U*^33^	*U*^12^	*U*^13^	*U*^23^
Li1	0.031 (3)	0.027 (3)	0.014 (3)	0.008 (2)	−0.001 (2)	−0.004 (2)
Li2	0.038 (4)	0.036 (4)	0.021 (3)	0.019 (3)	−0.010 (3)	−0.005 (3)
Li3	0.030 (2)	0.0212 (19)	0.0124 (19)	−0.0019 (16)	−0.0045 (17)	0.0002 (16)
S1	0.0196 (3)	0.0144 (3)	0.0120 (3)	0.00129 (19)	0.0004 (2)	−0.0002 (2)
S2	0.0209 (3)	0.0143 (3)	0.0088 (3)	−0.0003 (2)	−0.0007 (2)	0.0000 (2)
O1	0.0244 (9)	0.0204 (8)	0.0145 (8)	0.0041 (7)	−0.0042 (7)	−0.0033 (7)
O2	0.0284 (9)	0.0179 (9)	0.0261 (10)	−0.0009 (7)	−0.0054 (7)	0.0072 (7)
O3	0.0248 (9)	0.0192 (8)	0.0126 (8)	0.0044 (6)	0.0010 (7)	−0.0002 (7)
O4	0.0298 (9)	0.0212 (9)	0.0195 (9)	−0.0012 (7)	−0.0070 (7)	0.0025 (7)
N1	0.0288 (11)	0.0145 (9)	0.0133 (10)	0.0030 (8)	−0.0010 (8)	−0.0031 (8)
C1	0.0219 (13)	0.0309 (14)	0.0249 (14)	0.0058 (10)	0.0050 (11)	0.0002 (11)
F1	0.0226 (8)	0.0376 (9)	0.0362 (10)	0.0036 (6)	−0.0033 (7)	0.0029 (7)
F2	0.0379 (10)	0.0616 (13)	0.0549 (12)	0.0145 (9)	0.0075 (9)	−0.0308 (11)
F3	0.0339 (9)	0.0677 (14)	0.0532 (12)	0.0096 (9)	0.0184 (9)	0.0374 (11)
C2	0.0310 (13)	0.0187 (12)	0.0173 (12)	0.0000 (10)	0.0041 (11)	0.0028 (10)
F4	0.0324 (8)	0.0305 (8)	0.0337 (9)	−0.0125 (7)	0.0053 (7)	−0.0030 (7)
F5	0.0455 (10)	0.0399 (9)	0.0165 (8)	−0.0008 (7)	0.0135 (7)	−0.0035 (7)
F6	0.0452 (10)	0.0294 (9)	0.0418 (10)	0.0070 (7)	0.0111 (8)	0.0208 (8)
S3	0.0207 (3)	0.0140 (3)	0.0102 (3)	−0.0003 (2)	0.0020 (2)	−0.0006 (2)
S4	0.0196 (3)	0.0143 (3)	0.0090 (3)	−0.00059 (19)	−0.0006 (2)	0.0010 (2)
O5	0.0367 (10)	0.0172 (8)	0.0141 (8)	−0.0066 (7)	0.0034 (7)	−0.0030 (7)
O6	0.0297 (9)	0.0206 (9)	0.0129 (8)	0.0053 (7)	−0.0016 (7)	−0.0008 (7)
O7	0.0216 (8)	0.0219 (8)	0.0148 (8)	0.0032 (7)	−0.0004 (7)	−0.0040 (7)
O8	0.0336 (9)	0.0208 (8)	0.0123 (8)	−0.0018 (7)	−0.0010 (7)	0.0009 (7)
N2	0.0249 (10)	0.0152 (9)	0.0143 (10)	0.0029 (7)	−0.0021 (8)	0.0006 (8)
C3	0.0274 (13)	0.0244 (12)	0.0195 (13)	−0.0011 (10)	0.0074 (10)	−0.0037 (10)
F7	0.0470 (10)	0.0403 (9)	0.0162 (8)	−0.0102 (7)	0.0124 (7)	−0.0057 (7)
F8	0.0400 (10)	0.0579 (12)	0.0417 (11)	−0.0271 (9)	0.0201 (8)	−0.0218 (9)
F9	0.0543 (12)	0.0562 (13)	0.0499 (12)	0.0327 (10)	0.0257 (10)	0.0126 (10)
C4	0.0225 (12)	0.0198 (12)	0.0182 (12)	−0.0019 (9)	0.0020 (10)	−0.0005 (10)
F10	0.0370 (9)	0.0275 (8)	0.0366 (10)	−0.0109 (7)	0.0078 (7)	−0.0144 (7)
F11	0.0374 (9)	0.0285 (8)	0.0258 (8)	−0.0067 (7)	0.0066 (7)	0.0096 (7)
F12	0.0205 (7)	0.0323 (8)	0.0402 (10)	0.0021 (6)	−0.0004 (7)	0.0014 (7)
N3	0.0307 (12)	0.0214 (11)	0.0311 (13)	−0.0008 (10)	−0.0080 (10)	0.0037 (10)
C5	0.0284 (13)	0.0240 (13)	0.0177 (13)	−0.0068 (11)	−0.0007 (10)	0.0017 (10)
C6	0.0331 (14)	0.0303 (14)	0.0223 (14)	0.0057 (11)	0.0032 (11)	0.0059 (11)
N4	0.0486 (14)	0.0224 (11)	0.0157 (11)	−0.0041 (10)	0.0017 (10)	−0.0001 (10)
C7	0.0328 (14)	0.0217 (13)	0.0174 (13)	−0.0022 (10)	−0.0018 (11)	0.0041 (11)
C8	0.0313 (14)	0.0273 (14)	0.0236 (14)	−0.0007 (10)	0.0019 (11)	−0.0061 (11)

### Geometric parameters (Å, °)

**Table:**

Li1—O3	2.0473 (17)	S3—O5	1.4275 (18)
Li1—O1	2.0817 (17)	S3—O6	1.4370 (18)
Li1—O5	2.2678 (17)	S3—N2	1.576 (2)
Li2—O7	2.0247 (17)	S3—C3	1.838 (3)
Li2—O6	2.0831 (17)	S4—O8	1.4284 (18)
Li2—O2	2.3243 (18)	S4—O7	1.4326 (18)
Li3—O4	1.940 (5)	S4—N2	1.575 (2)
Li3—O8	1.942 (5)	S4—C4	1.831 (3)
Li3—N3	2.004 (5)	C3—F8	1.317 (3)
Li3—N4	2.006 (5)	C3—F9	1.319 (3)
S1—O2	1.4263 (18)	C3—F7	1.320 (3)
S1—O1	1.4347 (18)	C4—F11	1.321 (3)
S1—N1	1.573 (2)	C4—F10	1.323 (3)
S1—C1	1.844 (3)	C4—F12	1.323 (3)
S2—O4	1.4272 (19)	N3—C5	1.139 (4)
S2—O3	1.4317 (18)	C5—C6	1.455 (4)
S2—N1	1.574 (2)	C6—H61	0.9800
S2—C2	1.838 (3)	C6—H62	0.9800
C1—F1	1.318 (4)	C6—H63	0.9800
C1—F2	1.318 (4)	N4—C7	1.138 (4)
C1—F3	1.322 (3)	C7—C8	1.450 (4)
C2—F6	1.316 (3)	C8—H81	0.9800
C2—F5	1.323 (3)	C8—H82	0.9800
C2—F4	1.329 (3)	C8—H83	0.9800
			
O3^i^—Li1—O3	180.00 (3)	F2—C1—F3	109.3 (3)
O3^i^—Li1—O1	94.49 (7)	F1—C1—S1	109.02 (19)
O3—Li1—O1	85.51 (7)	F2—C1—S1	110.3 (2)
O3^i^—Li1—O1^i^	85.51 (7)	F3—C1—S1	110.43 (18)
O3—Li1—O1^i^	94.49 (7)	F6—C2—F5	109.4 (2)
O1—Li1—O1^i^	180.00 (9)	F6—C2—F4	109.5 (2)
O3^i^—Li1—O5^i^	89.99 (7)	F5—C2—F4	108.2 (2)
O3—Li1—O5^i^	90.01 (7)	F6—C2—S2	109.43 (18)
O1—Li1—O5^i^	89.90 (7)	F5—C2—S2	110.27 (18)
O1^i^—Li1—O5^i^	90.10 (7)	F4—C2—S2	110.04 (18)
O3^i^—Li1—O5	90.01 (7)	O5—S3—O6	118.77 (12)
O3—Li1—O5	89.99 (7)	O5—S3—N2	109.75 (11)
O1—Li1—O5	90.10 (7)	O6—S3—N2	115.79 (11)
O1^i^—Li1—O5	89.90 (7)	O5—S3—C3	103.57 (11)
O5^i^—Li1—O5	180.00 (8)	O6—S3—C3	104.46 (12)
O7^ii^—Li2—O7	180.00 (6)	N2—S3—C3	102.08 (12)
O7^ii^—Li2—O6^ii^	86.00 (7)	O8—S4—O7	117.44 (11)
O7—Li2—O6^ii^	94.00 (7)	O8—S4—N2	108.63 (11)
O7^ii^—Li2—O6	94.00 (7)	O7—S4—N2	115.75 (11)
O7—Li2—O6	86.00 (7)	O8—S4—C4	102.51 (12)
O6^ii^—Li2—O6	180.00 (8)	O7—S4—C4	105.05 (11)
O7^ii^—Li2—O2	91.11 (7)	N2—S4—C4	105.86 (11)
O7—Li2—O2	88.89 (7)	S3—O5—Li1	157.68 (11)
O6^ii^—Li2—O2	90.76 (7)	S3—O6—Li2	128.77 (11)
O6—Li2—O2	89.24 (7)	S4—O7—Li2	135.66 (11)
O7^ii^—Li2—O2^ii^	88.89 (7)	S4—O8—Li3	151.60 (18)
O7—Li2—O2^ii^	91.11 (7)	S4—N2—S3	125.01 (13)
O6^ii^—Li2—O2^ii^	89.24 (7)	F8—C3—F9	109.2 (3)
O6—Li2—O2^ii^	90.76 (7)	F8—C3—F7	108.1 (2)
O2—Li2—O2^ii^	180.00 (6)	F9—C3—F7	108.5 (2)
O4—Li3—O8	101.3 (2)	F8—C3—S3	111.08 (18)
O4—Li3—N3	102.0 (2)	F9—C3—S3	110.49 (19)
O8—Li3—N3	117.6 (2)	F7—C3—S3	109.38 (19)
O4—Li3—N4	116.6 (2)	F11—C4—F10	109.1 (2)
O8—Li3—N4	100.6 (2)	F11—C4—F12	108.9 (2)
N3—Li3—N4	118.1 (2)	F10—C4—F12	108.8 (2)
O2—S1—O1	118.69 (11)	F11—C4—S4	110.56 (18)
O2—S1—N1	110.18 (11)	F10—C4—S4	109.24 (17)
O1—S1—N1	115.64 (11)	F12—C4—S4	110.19 (17)
O2—S1—C1	103.54 (12)	C5—N3—Li3	168.4 (3)
O1—S1—C1	104.28 (12)	N3—C5—C6	179.6 (3)
N1—S1—C1	102.06 (12)	C5—C6—H61	109.5
O4—S2—O3	117.61 (11)	C5—C6—H62	109.5
O4—S2—N1	108.98 (11)	H61—C6—H62	109.5
O3—S2—N1	115.77 (11)	C5—C6—H63	109.5
O4—S2—C2	102.09 (12)	H61—C6—H63	109.5
O3—S2—C2	105.03 (11)	H62—C6—H63	109.5
N1—S2—C2	105.62 (12)	C7—N4—Li3	167.9 (3)
S1—O1—Li1	128.84 (11)	N4—C7—C8	179.5 (3)
S1—O2—Li2	157.89 (12)	C7—C8—H81	109.5
S2—O3—Li1	135.48 (11)	C7—C8—H82	109.5
S2—O4—Li3	152.09 (18)	H81—C8—H82	109.5
S1—N1—S2	125.29 (13)	C7—C8—H83	109.5
F1—C1—F2	109.5 (2)	H81—C8—H83	109.5
F1—C1—F3	108.2 (2)	H82—C8—H83	109.5
			
O2—S1—O1—Li1	93.14 (15)	O3^i^—Li1—O5—S3	139.2 (3)
N1—S1—O1—Li1	−41.19 (18)	O3—Li1—O5—S3	−40.8 (3)
C1—S1—O1—Li1	−152.37 (14)	O1—Li1—O5—S3	44.7 (3)
O3^i^—Li1—O1—S1	−141.25 (14)	O1^i^—Li1—O5—S3	−135.3 (3)
O3—Li1—O1—S1	38.75 (14)	O5—S3—O6—Li2	94.77 (15)
O5^i^—Li1—O1—S1	128.77 (14)	N2—S3—O6—Li2	−39.14 (18)
O5—Li1—O1—S1	−51.23 (14)	C3—S3—O6—Li2	−150.52 (14)
O1—S1—O2—Li2	−86.1 (3)	O7^ii^—Li2—O6—S3	−142.32 (14)
N1—S1—O2—Li2	50.5 (4)	O7—Li2—O6—S3	37.68 (14)
C1—S1—O2—Li2	159.0 (3)	O2—Li2—O6—S3	−51.26 (14)
O7^ii^—Li2—O2—S1	137.1 (3)	O2^ii^—Li2—O6—S3	128.74 (14)
O7—Li2—O2—S1	−42.9 (3)	O8—S4—O7—Li2	−144.13 (14)
O6^ii^—Li2—O2—S1	−136.9 (3)	N2—S4—O7—Li2	−13.6 (2)
O6—Li2—O2—S1	43.1 (3)	C4—S4—O7—Li2	102.77 (16)
O4—S2—O3—Li1	−143.85 (14)	O6^ii^—Li2—O7—S4	170.77 (16)
N1—S2—O3—Li1	−12.6 (2)	O6—Li2—O7—S4	−9.23 (16)
C2—S2—O3—Li1	103.50 (17)	O2—Li2—O7—S4	80.09 (16)
O1—Li1—O3—S2	−9.75 (15)	O2^ii^—Li2—O7—S4	−99.91 (16)
O1^i^—Li1—O3—S2	170.25 (15)	O7—S4—O8—Li3	115.4 (4)
O5^i^—Li1—O3—S2	−99.65 (16)	N2—S4—O8—Li3	−18.4 (4)
O5—Li1—O3—S2	80.35 (16)	C4—S4—O8—Li3	−130.1 (4)
O3—S2—O4—Li3	107.9 (4)	O4—Li3—O8—S4	−28.7 (5)
N1—S2—O4—Li3	−26.5 (4)	N3—Li3—O8—S4	81.5 (5)
C2—S2—O4—Li3	−137.8 (4)	N4—Li3—O8—S4	−148.9 (3)
O8—Li3—O4—S2	−19.3 (5)	O8—S4—N2—S3	155.10 (15)
N3—Li3—O4—S2	−141.1 (3)	O7—S4—N2—S3	20.5 (2)
N4—Li3—O4—S2	88.7 (4)	C4—S4—N2—S3	−95.43 (17)
O2—S1—N1—S2	−131.85 (16)	O5—S3—N2—S4	−133.72 (15)
O1—S1—N1—S2	6.2 (2)	O6—S3—N2—S4	4.1 (2)
C1—S1—N1—S2	118.67 (17)	C3—S3—N2—S4	116.90 (17)
O4—S2—N1—S1	154.01 (15)	O5—S3—C3—F8	−170.8 (2)
O3—S2—N1—S1	18.8 (2)	O6—S3—C3—F8	64.2 (2)
C2—S2—N1—S1	−96.96 (17)	N2—S3—C3—F8	−56.7 (2)
O2—S1—C1—F1	70.7 (2)	O5—S3—C3—F9	−49.4 (2)
O1—S1—C1—F1	−54.1 (2)	O6—S3—C3—F9	−174.4 (2)
N1—S1—C1—F1	−174.84 (18)	N2—S3—C3—F9	64.7 (2)
O2—S1—C1—F2	−49.6 (2)	O5—S3—C3—F7	70.1 (2)
O1—S1—C1—F2	−174.4 (2)	O6—S3—C3—F7	−54.9 (2)
N1—S1—C1—F2	64.9 (2)	N2—S3—C3—F7	−175.92 (17)
O2—S1—C1—F3	−170.5 (2)	O8—S4—C4—F11	−177.28 (17)
O1—S1—C1—F3	64.7 (2)	O7—S4—C4—F11	−54.0 (2)
N1—S1—C1—F3	−56.1 (2)	N2—S4—C4—F11	68.9 (2)
O4—S2—C2—F6	−58.6 (2)	O8—S4—C4—F10	−57.2 (2)
O3—S2—C2—F6	64.6 (2)	O7—S4—C4—F10	66.1 (2)
N1—S2—C2—F6	−172.51 (19)	N2—S4—C4—F10	−170.98 (17)
O4—S2—C2—F5	61.7 (2)	O8—S4—C4—F12	62.3 (2)
O3—S2—C2—F5	−175.04 (18)	O7—S4—C4—F12	−174.42 (17)
N1—S2—C2—F5	−52.2 (2)	N2—S4—C4—F12	−51.5 (2)
O4—S2—C2—F4	−178.96 (17)	O4—Li3—N3—C5	−56.2 (14)
O3—S2—C2—F4	−55.7 (2)	O8—Li3—N3—C5	−166.0 (12)
N1—S2—C2—F4	67.1 (2)	N4—Li3—N3—C5	73.1 (14)
O6—S3—O5—Li1	−88.8 (3)	O4—Li3—N4—C7	−157.1 (12)
N2—S3—O5—Li1	47.7 (4)	O8—Li3—N4—C7	−48.6 (14)
C3—S3—O5—Li1	156.1 (3)	N3—Li3—N4—C7	80.8 (13)

Symmetry codes: (i) −*x*+2, −*y*−1, −*z*+1; (ii) −*x*+2, −*y*, −*z*+1.
